# Molecular detection and antibiotic resistance pattern of extended-spectrum beta-lactamase producing *Escherichia coli* in a Tertiary Hospital in Enugu, Nigeria

**DOI:** 10.1186/s12941-019-0342-9

**Published:** 2019-12-12

**Authors:** Ifeyinwa N. Nwafia, Martin E. Ohanu, Samuel O. Ebede, Uchenna C. Ozumba

**Affiliations:** Department of Medical Microbiology, Faculty of Medical Sciences, College of Medicine, University of Nigeria, Ituku-Ozalla Campus, Enugu State, Nigeria

**Keywords:** Antimicrobial resistance, *Escherichia coli*, Extended-spectrum beta-lactamase, Molecular detection, Risk factors

## Abstract

**Background:**

The use of antibiotic agents in the treatment of infectious diseases has greatly contributed to the decrease in morbidity and mortality, but these great advances in treatment are being undermined by the rapidly increasing antimicrobial resistant organisms. Extended-spectrum beta-lactamases are enzymes hydrolyzing the beta lactam antibiotics, including third generation cephalosporins and monobactams but not cephamycins and carbapenems. They pose a serious global health threat and have become a challenge for health care providers. The aim of this research was to assess the prevalence of extended-spectrum beta-lactamase producing *Escherichia coli* in University of Nigeria Teaching Hospital Ituku-Ozalla Enugu and to detect the risk factors for acquisition of the resistant organism. To proffer advice on antibiotic stewardship in clinical practice and public health interventions, to curb the spread of the resistant organisms in the hospital.

**Results:**

Out of the 200 *E. coli* isolates, 70 (35.00%) were confirmed positive for extended-spectrum beta-lactamase production. Fifty-three (75.7%) were from hospital acquired infections. All the isolates were resistant to ampicillin, tetracycline and chloramphenicol while 68 (97.14%) of the 70 isolates were susceptible to imipenem. *Bla*_TEM_, *bla*_SHV_ and *bla*_TEM_ were detected in 66 (94%) of the 70 isolates. The ESBL *bla* genes detected were *bla*_CTX-M_ (n = 26; 37.14%), *bla*_TEM_ (n = 7; 10.00%), *bla*_SHV_ (n = 2; 2.86%), *bla*_CTX-M/TEM_ (n = 7; 10.0%), *bla*_CTX-M/SHV_ (n = 14; 20.0%) and *bla*_CTX-M/TEM/SHV_ (n = 10; 14.29%). The three *bla* genes were not detected in 4 (5.71%) of the isolates. Recent surgery, previous antibiotic and intensive care unit admission were the associated risk factors to infections caused by extended-spectrum beta-lactamase producing *E. coli*.

**Conclusion:**

There is a high rate of infections caused by extended-spectrum beta-lactamase producing *E. coli*. Recent surgery, previous antibiotic and intensive care unit admission were associated risk factors.

## Background

*Escherichia coli* (*E. coli*) is a common enteric organism and one of the predominant species in most bacterial infections [1]. Extended-spectrum beta-lactamase (ESBL) producing *E. coli* strains are causing global public health threats. They are resistant to several classes of antibiotics [2], which results in limited therapeutic options to treat the infections caused by these pathogens. The rapid increase in the spread of these antimicrobial resistant organisms, coupled with the unavailability of effective antimicrobial agents has made the World Health Organization (WHO) to warn against “post-antimicrobial era”, where people die because of common infections and minor injuries [3]. Extended-spectrum beta-lactamases (ESBLs) are plasmid-encoded enzymes and are easily transferred from one bacterium to another by horizontal gene transfer. Horizontal gene transfer by these plasmid exchanges between *E. coli* strains is a recognized source of rapid spread of antimicrobial resistant strains [4]. The most frequently detected and clinically important ESBLs belong to the Temoniera (TEM), sulfhydryl variable (SHV) and cefotaximase-Munich (CTX-M) families [5]. The burden of infections caused by these resistant strains is enormous. These strains are associated with a higher mortality rate, increased length of hospital stay and increased health costs [6, 7]. Owing to these consequences, coupled with the increasing incidence, it is important to carry out continuous surveillance on ESBL producing *E. coli* in the hospital. Therefore, this study was undertaken to investigate antibiotic resistance patterns, risk factors and molecular detection of *bla*_CTX-M_, *bla*_TEM_ and *bla*_SHV_ genes in *E. coli* in University of Nigeria Teaching Hospital Ituku-Ozalla, Enugu, with a view to providing comprehensive and reliable epidemiological information which will be used in the improvement of patient care and advice on antibiotic stewardship in clinical practice.

## Methods

### Study design and setting

This cross sectional study was conducted in Department of Medical Microbiology at the University of Nigeria Teaching Hospital (UNTH) Ituku-Ozalla, Enugu from January 2016 to May 2017. UNTH Ituku-Ozalla, Enugu is situated in Enugu state which is one of the five states in the south-east geopolitical zone of Nigeria. The hospital is a 450 bed tertiary healthcare institution with facilities for comprehensive pediatrics and adult healthcare services, including intensive care unit.

### Patient

The study involved 200 admitted patients in UNTH, Enugu. All consenting admitted adult (≥ 18 years) patients whose culture results were positive for *E. coli* within the study period were included in the survey. To calculate the number of samples required to estimate the prevalence of ESBL producing *E. coli* in the hospital, we used one-sample Z-test with an estimated prevalence of 11%, a confidence interval of 95% and maximum tolerable error of 10%. This gave a minimum sample size of 120 samples. The clinical samples analyzed were urine (106), pleural and peritoneal aspirate (24), blood (4), wound swabs (53) and cerebro-spinal fluids (13).

### Patient involvement

Patients were not directly involved, but their data were collected with a pretested structured questionnaire on their clinical records, after obtaining a written informed consent from them or their relatives. The questionnaire comprised four sections namely, demographics, history of present illness, past medical and co-morbidity history, devices utilization and past antibiotic used. The following data were then collected; ward admitted, age, gender, underlying disease, site of infection (from which *E. coli* was isolated), recent surgical procedure (within 3 months), immunosuppressant use within 1 month, parenteral nutrition and the presence of an indwelling urinary catheter and/or percutaneous tubes. Also previous antibiotic use (past 3 months) and the antibiotic taken were assessed. The investigator informed the patients and their relatives about their results and specific key infection prevention and control measures on how to prevent the spread of the resistant genes.

### Bacterial isolation and antibiotic susceptibility testing

All the samples collected were cultured on MacConkey and 5% sheep Blood agar plates (Oxoid Laboratories, Cambridge UK) and incubated aerobically at 37 °C for 24 h. All suspected isolates with growth characteristics of *E. coli* were subjected to standard bacteriological identification methods and confirmed with API 20E confirmation system (Biomerieux, Marcy-Etoile, France).

Antibiotic susceptibility testing with 17 antibiotic agents (ceftazidime, cefotaxime, amoxicillin plus clavulanic acid, aztreonam, gentamicin, amikacin, ciprofloxacin, ofloxacin, nitrofurantoin, piperacillin/tazobactam, chloramphenicol, ampicillin, tetracycline, ampicillin, ertapenem, imipenem and meropenem (Oxoid, Cambridge, UK) were performed on Muller Hinton agar plates by disc diffusion method. A maximum of five antibiotic discs were placed on each plate for each isolated *E. coli* strain and then incubated at 37 °C for 24 h, and the results recorded by measuring the inhibition zone diameter across the disc with a caliper and interpreted according to the Clinical and Laboratory Standard Institute guideline [8]. The quality control strains used were *E. coli* American type culture collection (ATCC) 25922 (ESBL negative strain) and *E. coli* ATCC 700603 (ESBL positive strain). *E. coli* isolates that were not susceptible to any of the third-generation cephalosporins were identified as potential ESBL producers and were then confirmed with chromogenic agar method. (Oxoid, Cambridge, UK). Seventy extended spectrum beta-lactamase producing *E. coli* confirmed isolates were immediately sub-cultured and preserved in a nutrient agar slant at 2–8 °C in a refrigerator and trypticase soy broth with 30% glycerol at − 70 °C in the freezer for subsequent molecular characterization.

### Molecular detection of the ESBL genes

Molecular detection of *bla*_CTX-M_, *bla*_TEM_ and *bla*_SHV_ were done by multiplex polymerase chain reaction (PCR). The extraction of the DNA was done with mini-prep kit (Jena Bioscience, Jena, Germany) according to manufacturer’s instructions. The primer used and their amplicon sizes were as shown in Table 1. The PCR amplification was performed in 20 µl reaction mixture containing the Solis Biodyne hot start Master mix (ready-to-load) containing 200 µM each deoxynucleoside triphosphates (dNTP), 2 mM MgCl_2_, 1× PCR Buffer, 2.0 units of TaqDNA polymerase, proof reading enzyme, 3 μl of DNA (10–200 ng), and sterile nuclease-free water was used to make up the volume of the reaction mixture. The thermal cycling was conducted in an Eppendorf thermal cycler (Nexus series) at an initial denaturation of 95 °C for 15 min, followed by 35 amplification cycles of 30 s at 95 °C; 30 s at 60 °C and 1 min at 72 °C. This was followed by a final extension step of 72 °C for 10 min. After amplification the product was separated on a 1.5% agarose gel electrophoresis and visualized by ethidium bromide staining. 100 base pair DNA ladders (Thermo Scientific) were used as DNA molecular weight standards.

**Table 1:** Primer sequences and their amplicon sizes

Primers	Nucleotide sequence	Base pair (bp)
TEM-F	GCGGAACCCCTATTTG	964
TEM-R	TCTAAAGTATATATGAGTAAACTTGGTCTGAC	964
SHV-F	TTCGCCTGTGTATTATCTCCCTG	854
SHV-R	TTAGCGTTGCCAGTGYTCG	854
CTX-M F	ATGTGCAGYACCAGTAARGTKATGGC	593
CTX-M R	TGGGTRAARTARGTSACCAGAAYCAGCGG	593

*n* Number

### Statistical analysis

All statistical analyses were performed using Statistical Package for Social Sciences (SPSS) computer software version 22. Descriptive analyses using percentages and frequencies were used for presence of antibiotic resistance pattern of extended-spectrum beta-lactamases and specific ESBL resistant genes. Continuous variables were compared with Chi square and multivariate logistic regression analysis to identify associated risk factors to ESBL producing *E. coli* infections. *P* < 0.05 was considered statistically significant.

## Results

During the study period, a total of 200 *E. coli* isolates were analysed from samples collected from patients with hospital 117 (58.5%) and community 83 (41.5%) acquired infections. Samples from female patients were 105 (52.5%) and males 95 (47.5%), with their ages ranging from 18 to 95 years old. Urine samples 106 (53.0%) had the highest frequency followed by wound swab 53 (26.5%), pleural and peritoneal aspirate 24 (12.0%), cerebrospinal fluid 13 (6.5%) and blood specimen 4 (2.00%). Seventy (35.0%) isolates were positive for extended spectrum beta lactamase production, 53 (75.7%) from hospital acquired infections and 17 (24.3%) from community acquired infections (Table 2).

**Table 2:** Sample distribution of ESBL positive and ESBL negative *Escherichia coli* from hospital and community acquired infections

Sample	HAI			CAI			Total
	ESBL (+)	ESBL (−)	Subtotal	ESBL (+)	ESBL (−)	Subtotal	
Urine	37	27	64	8	34	42	106(53.0)
Urine	5	28	33	5	15	20	53(26.5)
Pleural/Peritoneal fluid	3	8	11	3	10	13	24(12.0)
CSF	4	1	5	1	7	8	13(6.5)
Blood	4	0	4	0	0	0	4(2.0)
Total	53	64	117	17	66	83	200(100)

*HAI* hospital acquired infection, *CAI* community acquired infection, *+* positive, *−* negative, *CSF* cerebrospinal fluid

Results of the antimicrobial susceptibility testing are shown in Table 3. Low rates of sensitivity were found against third generation cephalosporins {Cefotaxime 5 (7.1%), ceftazidime 7 (10.00%), ceftriaxone 10 (14.2%)} and aztreonam 3 (4.2%). Among the quinolones, sensitivity to ciprofloxacin and ofloxacin were 15 (21.4%) and 26 (37.1%) respectively. In aminoglycosides, sensitivity to amikacin 32 (45.7%) was slightly higher than gentamicin 21 (30.00%). Slightly more than half 39 (55.71%) of the isolates were sensitive to nitrofurantoin. Sensitivity was highest with imipenem, 68 (97.1%), followed by meropenem 65 (92.8%), ertapenem 65 (92.86%) and piperacillin/tazobactam 42 (60.0%). None of the isolates were sensitive to ampicillin, chloramphenicol and tetracycline.

**Table 3:** Antibiotic susceptibility pattern of ESBL producing *Escherichia coli*

Antibiotics	S (%)	I (%)	R (%)
Amikacin	32 (45.71)	6 (8.71)	32 (45.43)
Ampicillin	0 (0.00)	0 (0.00)	70 (100.00)
Amoxicillin–clavulanic acid	25 (35.71)	7 (10.00)	38 (54.29)
Aztreonam	3 (4.29)	0 (0.00)	67 (95.71)
Ceftriaxone	10 (14.29)	2 (2.86)	58 (82.86)
Cefotaxime	5 (7.14)	2 (2.86)	63 (90.00)
Ceftazidime	7 (10.00)	2 (2.86)	61 (87.14)
Chloramphenicol	0 (0.00)	0 (0.00)	70 (100.00)
Ciprofloxacin	15 (21.43)	3 (4.29)	52 (74.29)
Ertapenem	65 (92.86)	0 (0.00)	5 (7.14)
Gentamicin	21 (30.00)	4 (5.71)	45 (64.29)
Imipenem	68 (97.14)	1 (1.43)	1 (1.43)
Meropenem	65 (92.86)	1 (1.43)	4 (4.29)
Nitrofurantoin	39 (55.71)	4 (5.71)	27 (38.57)
Ofloxacin	26 (37.14)	0 (0.00)	44 (62.86)
Piperacillin/tazobactam	42 (60.00)	4 (5.71)	24 (34.29)
Tetracycline	0 (0.00)	0 (0.00)	70 (100.00)

*S* sensitivity, *I* intermediate, *R* resistant

**Fig. 1:**
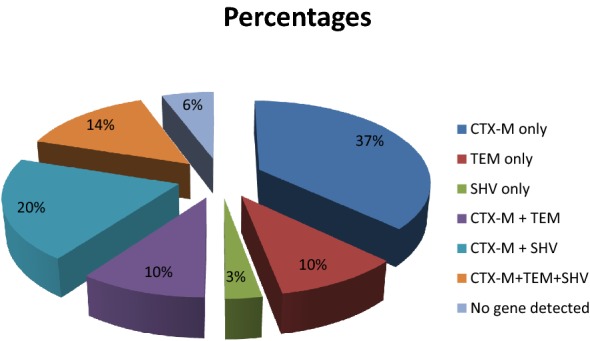
Distribution of SHV, TEM and CTX-M genes among the positive isolates. ESBL genes detected in *Escherichia coli* isolates from different samples by multiplex polymerase chain reaction. The following genes were detected. *Bla*_CTXM_ only 37%, *Bla*_TEM_ only 10%, *Bla*_SHV_3%, *Bla*_CTX-M+SHV_ 20%, *Bla*_CTXM+TEM_ 10%, *Bla*_CTX+TEM+SHV_ 14%

Sixty-six of the phenotypically confirmed ESBL producers contained at least one ESBL gene with no gene seen in 4 of the isolates. The *bla*_CTX-M_ was the predominate gene seen (Fig. 1).

The most associated risk factors to infections caused by ESBL producing *E. coli* was previous antibiotics use in the past 3 months (*P *= 0.006), intensive care unit admission (*P *= 0.04), recent surgery (*P *= 0.015) (Table 4).

**Table 4:** Multivariate analysis for risk factors associated with infections by ESBL producing *Escherichia coli*

Risk factor	Wald Chi-Square	OR	*P* value
Female	1.464	1.439	0.226
Old age (60 years)	1.324	0.681	0.250
Recent surgery	6.131	2.446	0.013
Previous antibiotic (≤ 3 months)	7.461	1.948	0.006
Beta-lactam	9.850	1.555	0.002
Aminoglycosides	0.831	0.484	0.362
Fluoroquinolones	3.456	0.455	0.063
Surgical ward	0.064	0.976	0.806
ICU ward	3.502	1.432	0.041
Medical ward	0.511	0.788	0.475
Orthopedic ward	2.784	1.563	0.095
Gynaecology ward	0.933	0.872	0.334
Post natal ward	0.006	0.989	0.939
Urinary Catheter	1.661	2.002	0.197
Nasogastric tubes	1.961	0.789	0.161
Central venous lines	0.181	1.290	0.671
Ventilator	1.245	2.142	0.264
Arterial lines	0.323	0.538	0.570
Endotracheal tube	0.048	1.117	0.827
Bronchoscopic tube	1.478	0.574	0.224
< 7 days stay in hospital	2.228	0.487	0.136
7–14days stay in hospital	0.537	1.154	0.464
> 14days stay in hospital	3.065	1.412	0.080

*OR* odd ratio

## Discussion

In this cross-sectional study, urine specimens accounted for the majority of *E. coli* positive specimens analysed. This fact is not surprising because urinary tract infections (UTI) have been established to be one of the commonest hospital-acquired infections in Nigeria and other parts of the world [9–11].

Our study revealed a high rate (70/200, 35.0%) of ESBL producing *E. coli* among hospitalized patients in Enugu, three times the rate reported in an earlier study (11.9%) in the same hospital in 2009 [12]. This increase within 8 years maybe due to unchecked antibiotics consumption especially beta-lactam antibiotics by the patients and poor infection prevention control measures. This finding is in agreement with what was reported in studies done in Bucharest (35.0%) [13] and Zaria (34.3%) [14]. Lower rates were reported in the South Eastern (16.0%) [15] and South Western (20.0%) [16] parts of Nigeria. This shows that there are wide geographical variations in rates and even within the same hospital over time as seen in the study. The rate of ESBL producing *E. coli* was significantly higher in hospital acquired infections. Although majority of the samples were gotten from patients with hospital acquired infection, but many studies have also reported higher rates in hospital acquired infections compared to community acquired infections [17, 18]. This maybe because of increase in the use of antibiotics and poor infection prevention and control measures.

The co-resistance seen in tetracycline, chloramphenicol and ampicillin has also been reported in other studies [19–22]. These drugs are cheap and easily available so they are easily abused by the patients. The highest rate of resistance to available and easily affordable antimicrobial agents is of great concern. Nigeria does not have a strong regulated antibiotic prescription system, so antibiotics are easily purchased over the counter even from non-licensed dispensers, making them to be easily abused. Third generation cephalosporins, quinolones and aminoglycosides showed very poor activities. High rates of co-resistance to these antibiotics were reported in earlier studies [22–24]. Extended-spectrum beta-lactamase producing bacteria are known to carry genes conferring resistance to several non-beta lactam antibiotics and this would partly explain the high resistance rates to these drugs seen in this study. The resistance rates to third generation cephalosporins (ceftazidime 87.1%, ceftriaxone 82.8% and cefotaxime 90.0%) reported in this study were higher than the previous rates reported in the same hospital by Iroha et al. [12]. They reported 28.1% in ceftazidime, ceftriaxone 34.9% and cefotaxime 22.5%. This highlights the increase in the spread of these resistance isolates in the hospital. Higher rates than what were obtained from this study have also been reported by Wani et al. [25] in India. There is superiority of local resistance data over regional, national or international data in the management of patients in any locality as it raises awareness to current resistance profile. Moderate resistances were seen in piperacillin/tazobactam and nitrofurantoin. Nitrofurantoin is a suitable, effective, and cheap alternative drug in the treatment of ESBL-producing *E. coli* related lower UTI [26]. Fortunately, carbapenems were found to be the most effective antibiotics against the ESBL producing *E. coli*, with imipenem being the most sensitive [27–29]. Carbapenems should be used with cautions because the overuse in the treatment of infections caused by ESBL producing organisms will gradually lead to acquisition and development of carbapenem resistant strains. There is a need to use alternatives such as nitrofurantoin and piperacillin/tazobactam as other options. This finding will help to guide development of effective antimicrobial stewardship and restriction policies.

Knowledge of the risk factors will help in early identification of patients likely to be infected with these organisms. This also is an important step in the prevention of the spread of resistant organisms among hospitalized patients, and visitors or relatives of the patients from their community. Control of the hospital spread will definitely have a role in minimising community spread of these resistant organisms. There is an association between previous exposures to antibiotics (especially beta-lactams) in the past 3 months to ESBL *E. coli*. This is consistent with previous studies showing that consumption of beta-lactams and fluoroquinolones are risk factors for predispositions to ESBL producing enterobacteriaceae carriage [30, 31]. Antibiotics misuse and abuse together with lack of knowledge of antibiotic resistance may be one of the major reasons for the increase in antibiotic resistance. There is a need to educate the prescribers and patients on rational and judicious use of antimicrobial agents. Other risk factors observed were admission in the intensive care unit and recent surgery. There are variations in the risk factors reported in different geographical locations. In Iran, Fatemeh et al. [9] reported recent surgery, prolonged antibiotic use, as the risk factors to infection caused by ESBL producing organisms.

This study confirmed that *bla*_CTX-M_ gene is the most common ESBL type harbored by *E. coli* strains in our hospital. This has been reported in other studies done in Nigeria [32–34]. This is because the plasmid carrying *bla*_CTX-M_ genes are known to carry other genes conferring resistance to several antibiotics. Secondly, the location of different resistance genes on single replicon leads to co-selection and may have contributed to the dissemination [35]. Yahaya et al. [27], in Maiduguri reported *bla*_SHV_ (36.4%) as the predominant gene followed by *bla*_TEM_ (31.4%) and *bla*_CTX-M_ (27.3%). In Bangladesh, Yesmin et al. [36] reported *bla*_TEM_ (50.5%) as the predominant gene followed by *bla*_CTX-M_ (46.7%) and *bla*_SHV_ (18.7%). All these studies confirmed that gene predominance varied between regions and locations and to a large extent determine the resistance profiles of the organisms in the locality. It was also noticed in this study that some of the bacteria possessed multiple genes as had been established in some earlier studies [15, 37].

To the best of our knowledge, this is the first study that did a molecular study of ESBL genes in our institution. This knowledge will guide clinicians in developing antibiotics guidelines and policies, aimed at curbing the spread of ESBL *E. coli*. Further research on whole genomic sequencing is advocated to fully understand the epidemiology behind the spread of these organisms. We should be a step ahead in the management of multidrug resistant organisms in order to mitigate the evolution of the superbug that can defy all known antibiotics.

## Conclusion

This study demonstrated a high prevalence (35.0%) of extended spectrum beta lactamase producing *E. coli* in our hospital. There is a need for urgent interventions, including regular surveillance aimed at curbing the spread of extended spectrum beta lactamase producing *E. coli*, development of effective hospital antibiotics policies and effective infection prevention and control measures.

## Data Availability

The datasets used and/or analysed during this study are available from the corresponding author on reasonable request.

## Abbreviations

ATCC: American type culture collection
AOR: adjusted odds ratio
API: analytic profile index
CTX-M: cefotaximase-Munich
CI: confidence interval
dNTP: deoxynucleoside triphosphates
ESBL: extended-spectrum beta-lactamase
PCR: polymerase chain reaction
SHV: sulfhydryl variable
SPSS: Statistical Package for Social Sciences
TEM: Temoniera
UNTH: University of Nigeria Teaching Hospital
UTI: urinary tract infection
WHO: World Health Organization
